# Croton tiglium essential oil compounds have anti-proliferative and pro-apoptotic effects in A549 lung cancer cell lines

**DOI:** 10.1371/journal.pone.0231437

**Published:** 2020-05-01

**Authors:** Qing-lin Niu, Hui Sun, Chao Liu, Juan Li, Chang-xu Liang, Rui-rui Zhang, Fu-rong Ge, Wei Liu

**Affiliations:** 1 Shandong Provincial Key Laboratory of Fruit Tree Biotechnology Breeding, Shandong Institute of Pomology, Tai’an, Shandong, China; 2 Key Laboratory of Novel Food Resources Processing, Ministry of Agriculture and Rural Affairs/Key Laboratory of Agro-Products Processing Technology of Shandong Province/Institute of Agro-Food Science and Technology, Shandong Academy of Agricultural Sciences, Jinan, Shandong, China; 3 Taian Traditional Chinese Medicine Hospital, Tai’an, Shandong, China; 4 School of Water Conservancy and Environment, University of Jinan, Jinan, Shandong, China; Duke University School of Medicine, UNITED STATES

## Abstract

As a traditional Chinese medicine, *Croton tiglium* has the characteristics of laxative, analgesic, antibacterial and swelling. This study aimed to analyze the chemical composition of *C*. *tiglium* essential oil (CTEO) extracted from the seeds of *C*. *tiglium* and its cytotoxicity and antitumor effect *in vitro*. Supercritical CO_2_ fluid extraction technology was used to extract CTEO and the chemical constituents of the essential oil were identified by comparing the retention indices and mass spectra data taken from the NIST library with those calculated based on the C7-C40 *n*-alkanes standard. *In vitro* cytotoxicity of the CTEO was assessed against cancer cell lines (A549) and the human normal bronchial epithelial cells (HBE) using the CCK-8 assay. Proliferation was detected by colony formation experiments. Wound scratch and cell invasion assays were used to detect cell migration and invasion. Levels of apoptotic markers, signaling molecules, and cell cycle regulators expression were characterized by Western blot analysis. As the results, twenty-eight compounds representing 92.39% of the total oil were identified in CTEO. The CTEO has significant antitumor activity on A549 cancer cells (IC_50_ 48.38 μg/mL). *In vitro* antitumor experiments showed that CTEO treatment significantly inhibited the proliferation and migration of A549 cells, disrupted the cell cycle process, and reduced the expression levels of cyclin A, cyclin B and CDK1. CTEO can also reduce mitochondrial membrane potential, activate caspase-dependent apoptosis pathway, and finally induce apoptosis. CTEO may become an effective anti-cancer drug and will be further developed for cancer treatment.

## Introduction

Lung cancer is the most common and lethal cancer worldwide, especially in developing countries [1]. It is estimated that 1,540,050 cases of lung cancer occurred in 2018 accounting for a quarter of deaths in the United States [2]. Among all lung cancer patients, non-small-cell lung cancer (NSCLC) is the major type and accounted for about 80–85% [3]. NSCLC patients show high metastasis potential, and approximately 70% patients have metastases to regional lymph nodes or to distant sites upon the initial detection of cancer [4]. In addition, the vast majority of patients are diagnosed at a late stage [5]. Despite advances in treatments of NSCLC, prognosis remains a challenging aspect of this uncontrolled systemic disease.

Plant essential oils are extracted commonly from fruits, leaves, branches, and seeds of aromatic plants [6]. Due to the strong toxicological effect of the chemical synthetic products, the components of natural essential oil are gaining increasing interest and frequent presence in studies investigating their potential functional utility [7, 8]. Essential oils has anti-inflammatory, antibacterial, anti-tumor, anti-oxidation and other functions and are abundantly used in indigenous medicines, food flavoring, drug and cosmetic industries [9–11]. About 300 plant essential oils are crucial in agricultural, cosmetic, food, and health industries.

As one genus of the Euphorbiaceae family, *Croton* consists of approximately 1300 species which are widely distributed in tropical and sub-tropical regions [12]. *C*. *tiglium* is one of the genus *Croton* and its seeds are well known as “Badou” in mainland China and utilized widely to treat gastrointestinal disorders, intestinal inflammation, rheumatism, headache, peptic ulcer, and visceral pain [13]. In 1963, the tumor-promoting principles of *C*. *tiglium* seeds were reported by Van Duuren [14]. After that, many bioactive phorbol esters were isolated and evaluated from this species. The major constituent, 12-O-tetradecanoylphorbol-13-acetate (TPA), has been used widely in biochemical experiments as standard tumor-promoting agent [15, 16]. However, the chemical composition of the plant directly results in the uses and treatments of different diseases. Except phorbol esters, diverse types of diterpenes were isolated and evaluated from this species based on the previous studies investigating [17, 18], and many of them exhibited remarkable anticancer activity and inhibition vessel formation in zebrafish [19, 20].

Previous reports showed that the *C*. *tiglium* essential oil (CTEO) had purgative, analgesic, antimicrobial, and inflammatory properties [13, 21]. There is an abundance of oleic acid, linoleic acid, and eicosenoic acid in a methyl-esterified sample of obtained by reflux method [22]. Although many chemical constituents of *C*. *tiglium* have obvious anti-tumor effects, it is not clear whether the CTEO (the low polarity fraction) also has the same effects, thus, the objective of this study is to explore the chemical composition of CTEO prepared by CO_2_ supercritical fluid extraction, as well as its potential anti-cancer activities and related molecular mechanisms.

## Materials and methods

### Plant materials

**Fig 1:**
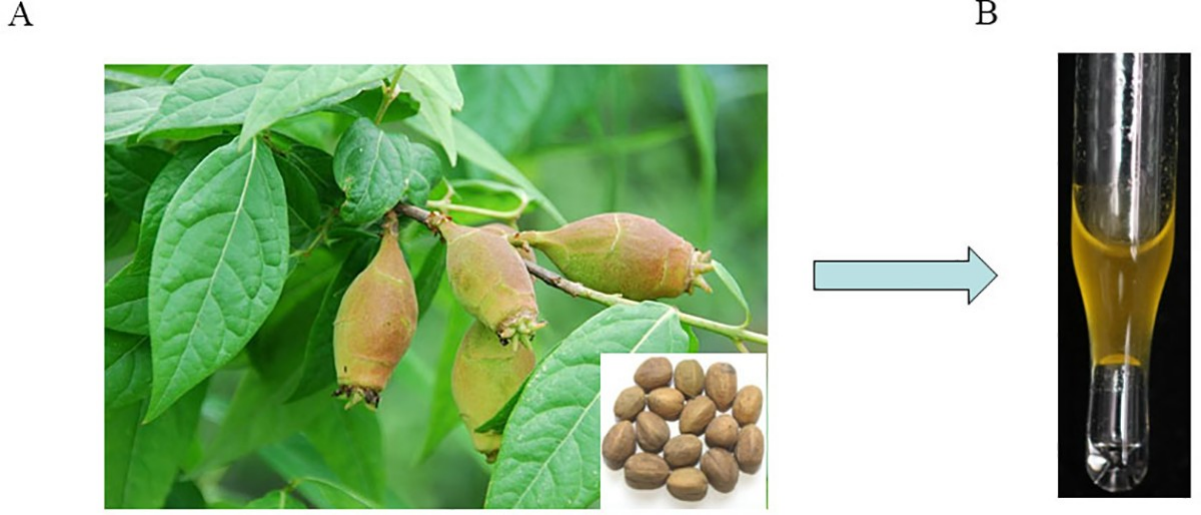
Morphological observation of *C*. *tiglium* fruits (A) and essential oil extracted by supercritical CO_2_ extraction (B).

*C*. *tiglium* fruits (brown, ellipsoidal with 6 to 7 mm in diameter) were purchased in the Chinese Herbal Medicine Market in the city of Bozhou, Anhui province, China in May, 2018. Prof. Chenggang Shan who comes from the institute of agro-food science and Technology, Shandong Academy of Agriculture Sciences (IAFST, SAAS), Jinan, China identified the species. A specimen (No.18-05-05) (Fig 1A) was deposited at the Laboratory of Bioactive Substances & Functional Foods, IAFST, SAAS.

### Essential oil extraction by supercritical fluid extraction

The purchased *C*. *tiglium* fruits (1.0 kg) are placed in the shade to dry and then ground into powder through a 40-mesh screen (450 μm), following extracted with CO_2_ in a supercritical extraction vessel (25 MPa, 35°C). The essential oil (Fig 1B) was obtained after 30 min static extraction following 30 min dynamic extraction with a flow rate of 2 L CO_2_/min. The SFE-CO_2_ extracts were collected in opaque bottles, weighed, and stored in a refrigerator at 4°C for the next experiment.

### GC/MS analysis

GC-MS analysis of CTEO was performed on an Agilent 6890B GC coupled with a 5977A mass selective detector (MSD; Agilent, Santa Clara, CA, USA). An HP-5MS capillary column (30 m × 0.25 mm, 0.25 μm film thickness; Restek Corporation, Bellefonte, PA, USA) was used. Helium was used as the carrier gas at 1 mL/min with the following temperature program: initial temperature at 100°C, increased to 200°C at the rate of 6°C/min, then 4°C/min up to 300°C and held for 10 min, for a total run of 51.7 min. The CTEO samples (1 μL) were injected at a temperature of 280°C with a split ratio of 1/70 over 1 min. An electron impact ionization system with ionization energy of 70 eV and electron ionization spectra with a mass scan range of 30–500 *m/z* was used.

### Identification of the essential oil components

Identification of the components of CTEO was conducted as described by Zhang *et al*. [22, 23], by comparing the mass spectra of the CTEO components with those from the NIST 08 mass spectra libraries and by comparing the calculated experimental GC retention indices determined using a mixture of a homologous series of normal alkanes from C7 to C40 in hexane under the same conditions described above with the GC retention indices reported in the NIST Standard Reference Database (NIST Chemistry WebBook, 2014, http://webbook.nist.gov/chemistry/). The percentage ratios of the CTEO components were computed using the normalization method of the GC/FID peak areas.

### Cell culture

A549 and HBE cells were purchased from the Cell Bank of Type Culture Collection of the Chinese Academy of Sciences (Shanghai, China). A549 cells and HBE cells were cultured in RPMI-1640 (HyClone) containing 10% foetal bovine serum (Gibco) and 100 U/mL penicillin-streptomycin (HyClone). All cells were cultured at 37°C containing 5% CO_2_ humidified conditions.

### CCK-8 assays

CCK-8 Kit was used to detect the cell viability. In brief, 5000 cells per wells were seeded into 96-well plates for 24 h. Following treatment with different doses of CTEO for 24, 48 and 72 h, 10 μL CCK-8 solution was added to each well, and the cells were incubated for additional 2 h at 37°C. The absorbance at 450 nm was assessed with a microplate reader (Bio Rad, Hercules, CA, USA) in the plate. The growth inhibition curve and half-maximal inhibitory concentration value were obtained from CCK-8 viability curves using GraphPad Prism software.

### Wound scratch assay

Wound scratch assay was used to assess cancer cell migration capability. For wound healing assays, cells were seeded into 6-well plates and incubated, and the cells were scratched with a 200 μL sterile pipette tip when cells reached 90% monolayer confluent. The cancer cells were subsequently treated with different concentrations of CTEO and photographed with an inverted microscope (Olympus Corp, Tokyo, Japan) at different time points to observe the distance of cell migration. Each experiment was repeated in triplicate.

### Cell invasion assays

For the invasion assay, the 24-well Transwell chamber were pre-coated with 70 μL diluted matrigel (1:2 dilutions with serum-free medium) in the upper chamber and placed in a 37 incubator for coagulation. The cancer cells (1×10^5^ cells/well) were resuspended in 5% FBS RPMI-1640 medium, and were treated with CTEO or alone, and seeded in the upper chamber. The lower chamber was placed in RPMI-1640 containing 20% FBS at 37°C in 5% CO_2_. After 24 h incubation, the non-invasive cells on the upper chambers were removed via gentle scraping, and those cells attached to the lower compartment were fixed with 4% paraformaldehyde and stained with crystal violet for 15–20 min at room temperature. Then, the upper chamber was washed with PBS twice. Randomly select different fields of view to take pictures under an inverted microscope (Olympus Corporation, Tokyo, Japan).

### Colony formation assay

A549 cells were seeded in 6 cm cell culture dishes (1000 cells). After 7 days, the cells were treated with various concentrations of CTEO for one week. The compounds were removed and washed with PBS per wells. Then, the cells were fixed with 4% paraformaldehyde at room temperature for 20 min, stained with Giemsa and photographed with a camera.

### Cell apoptosis analysis

Cell apoptosis assay was conducted using an Annexin V- FITC/PI Apoptosis detection kit (Beyotime, Nantong, China) and analyzed the apoptotic rate by flow cytometry. The cells were treated with different concentration CTEO for 48h. A certain number of cells were collected and resuspended in 195 μL Annexin V-FITC binding buffer, followed by the addition of 5 μL of annexin v-FITC and 10 μL of PI to mix. Then, the samples were incubated at room temperature for 30 min in the dark and assayed by flow cytometry (FACSCalibur, BD Biosciences).

### Mitochondrial membrane potential (ΔΨm) assay

The changes in mitochondrial membrane potential (MMP) was determined by the classical JC-1 kit (Beyotime, Nantong, China). The assay method was performed following the manufacturer’s instructions. A549 cells were treated with the aforementioned method. After 48 h, cells were collected in RPMI 1640 medium and 1 mL JC-1 working solution was added; then, the samples were incubated at 37°C for 20 min. Next, the staining cells were imaged using a confocal microscope (Nikon Corp, Tokyo, Japan).

### Western blot analysis

A549 cells were treated with different concentration CTEO for 48 h. After washing with ice-cold PBS, the cells were collected and lysed using RIPA lysis buffer with a protease inhibitor (Beyotime, Nantong, China). The cell lysates were subsequently centrifuged at 12000 g at 4°C for 15 min, and the proteins concentration were quantified by a BCA Protein Assay Kit (Beyotime, Nantong, China). About 50 μg proteins were subjected to 10% or 12% SDS-PAGE, and subsequently transferred onto PVDF membranes. The membranes were blocked with TBST containing 5% nonfat milk for 2 h at room temperature. Then, the blocked membranes were incubated overnight at 4°C with primary antibodies: rabbit polyclonal antibody to PCNA (1:1000; GB11010-1, Servicebio Technology), Cyclin A (1:1000; 91500S, Cell Signaling Technology), Cyclin B (1:500; GB11255, Servicebio Technology), CDK1 (1:500; GB11398, Servicebio Technology), P21 (1:500; BS1269, Bioworld Technology), p-FAK (1;500; BS4617,Bioworld Technology), FAK (1:1000; 12636-1-AP, Proteintech Technology), Bax (1:1000; GB11007, Servicebio Technology), BcL-2 (1:1000; BS1511, Bioworld Technology), cleaved caspase-3 (1:1000; 9661, Cell Signaling Technology), cleaved caspase-9 (1:1000; 7237, Cell Signaling Technology), cleaved PARP (1:1000; 56255,Cell Signaling Technology). Mouse polyclonal antibody to cytochrome C (1:1000; 66264-1-Ig, Proteintech Technology), GAPDH (1:1000; GB12002, Servicebio Technology) followed by incubation with secondary antibody for HRP-conjugated Affinipure Goat Anti-Mouse IgG(H+L) (1:1000; SA00001-1, Proteintech Technology) and HRP-conjugated Affinipure Goat Anti- rabbit IgG(H+L) (1:1000; SA00001-2, Proteintech Technology) at room temperature for 2 h with rocking. Then the proteins were detected using an ECL kit in Chemiluminescence imaging system. The results were normalized to GAPDH using ImageJ software.

### Statistical analysis

All experiments were repeated at least three times. The result were expressed as the mean ± standard deviation (SD) error of the mean. T test between groups were assessed by two-tailed Student. *P<0*.*05* was considered to indicate a statistically significant difference.

## Results

### Identification of the chemical constituents of CTEO

The CTEO extracted by SFE-CO_2_ was faint yellow oil (Fig 1B) with a yield of 2.51 ± 0.02% (w/w) in view of the dry weight of *C*. *tiglium* fruits. In order to determine volatile composition, the CTEO was analysed by GC-MS. As shown in Table 1 and S1 Fig, twenty-eight compounds were identified which is accounting for 92.39% of the total oil. The foremost compounds were 17-Octadecynoic acid at 36.73% followed by Tetradecanoic acid (8.49%), 17-Octadecynoic acid methyl ester (8.17%), *n*-Hexadecanoic acid (6.45%), *n*-Decanoic acid (5.28%), Linoleic acid ethyl ester (4.37%), and i-Propyl 9-octadecenoate (4.19%). Fatty acid and related esters, which consists of 17 compounds, are the main component of the CTEO.

**Table 1:** Chemical composition of CTEO by supercritical CO_2_ fluid extraction.

Num	RT^a^	RI_lit_^b^	RIca^c^	Compound	Formula	%
1	6.72	1191	1191	1-Dodecyne	C_12_H_22_	0.35
2	7.161	1317	1316	2,4-Decadienal	C_10_H_16_O	0.54
3	8.477	1373	1386	*n*-Decanoic acid	C_10_H_20_O_2_	5.28
4	8.912	1409	1409	1a,2,3,4,4a,5,6,7b-octahydro-1,1,4,7-tetramethyl-, 1H-Cycloprop[e]azulene	C_15_H_24_	0.24
5	12.105	1475	1475	Undecanoic acid	C_11_H_22_O_2_	2.54
6	12.168	1477	1477	*γ*-Himachalene	C_15_H_24_	0.21
7	15.91	1768	1782	Tetradecanoic acid	C_14_H_28_O_2_	8.49
8	18.244	1926	1914	Hexadecanoic acid methyl ester	C_17_H_34_O_2_	0.25
9	19.389	1968	1977	*n*-Hexadecanoic acid	C_16_H_32_O_2_	6.45
10	19.492	1993	1983	Hexadecanoic acid, ethyl ester	C_18_H_36_O_2_	0.66
11	21.06	2063	2067	9-Octadecen-1-ol, (Z)-	C_18_H_36_O	0.24
12	21.34	2092	2082	9,12-Octadecadienoic acid (Z,Z)-, methyl ester	C_19_H_34_O_2_	0.95
13	21.466	2091	2089	9-Octadecenoic acid (Z)-, methyl ester	C_19_H_36_O_2_	0.36
14	22.639	2151	2150	17-Octadecynoic acid, methyl ester	C_19_H_34_O_2_	8.17
15	22.771	2162	2157	Linoleic acid ethyl ester	C_20_H_36_O_2_	4.37
16	23.406	2199	2190	17-Octadecynoic acid	C_18_H_32_O_2_	36.73
17	23.469	2192	2193	i-Propyl 9-octadecenoate	C_21_H_40_O_2_	4.19
18	26.524	1849	1853	Z-(13,14-Epoxy)tetradec-11-en-1-ol acetate	C_16_H_28_O_3_	1.20
19	27.823	2393	2422	Gibberellic acid	C_19_H_22_O_6_	0.66
20	29.334	2010	2002	9-Octadecenal	C_18_H_34_O	0.64
21	31.737	2651	2633	Fumaric acid, 4-octyl dodec-2-en-1-yl ester	C_24_H_42_O_4_	0.60
22	32.567	2655	2679	1-Hydroxy-3-methoxypropan-2-yl octadeca-9,12-dienoate	C_22_H_40_O_4_	3.11
23	32.641	2707	2684	9,12-Octadecadienoic acid, 2-hydroxy-1-(hydroxymethyl)ethyl ester	C_21_H_38_O_4_	1.56
24	36.126	2508	2484	13-Docosenoic acid, methyl ester	C_23_H_44_O_2_	0.35
25	40.125	2139	2144	cis-Vaccenic acid	C_18_H_34_O_2_	0.39
26	41.487	3200	3217	β-Sitosterol	C_29_H_50_O	0.91
27	42.506	3277	3282	5-Ethyl-6-methylheptan-2-yl)-3-methoxy-10,13-dimethyl-2,3,4,7,8,9,10,11,12,13,14,15,16,17-tetradecahydro-cyclopenta[a]phenanthrene	C_30_H_52_O	2.95
28	49.973	3696	3669	Oleyl oleate	C_36_H_68_O_2_	0.67
Total content of identified compounds						92.39

^a^Retention time

^b^Retention index taken from NIST

^c^Retention index experimentally calculated based on the C7-C40 *n*-alkanes standard

### CTEO inhibits the viability of A549 lung cancer cells

**Fig 2:**
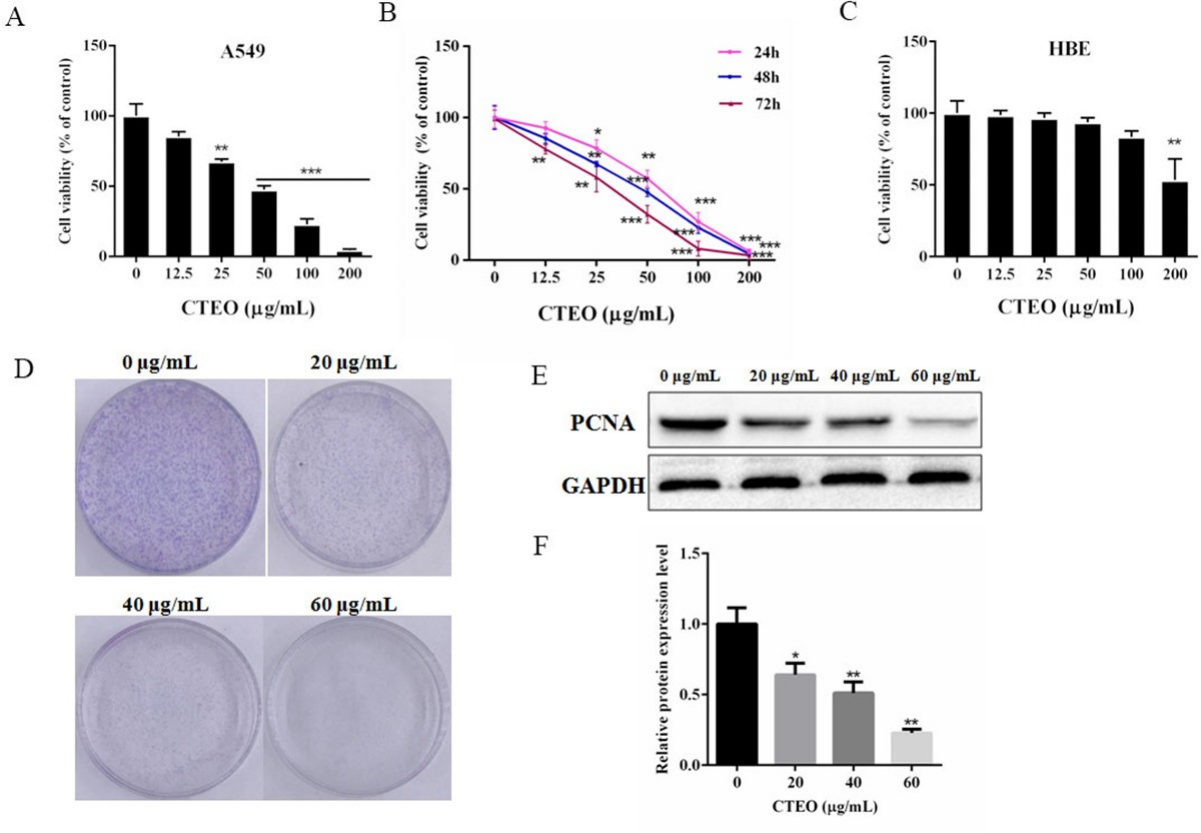
CTEO inhibits the viability of A549 lung cancer cells. A.The viability of A549 cells following 48 h of CTEO treatment was detected by CCK-8 assay. B. A549 cells were cultured with different concentrations (0–200 μg/mL) of CTEO for varied time points). Cell viability was determined by CCK-8 assay. C. The viability of HBE cells following 48 h of CTEO treatment was detected by CCK-8 assay. D. The colony formation of A549 cells was determined with colony formation assay after exposure to different concentrations of CTEO (0, 20, 40 and 60 μg/mL) and colonies were allowed to grow for 10 days. E, F. Western blotting analysis of PCNA protein expression after CTEO treatment, and quantification. Values represent mean ± SD of three independent experiments. *P < 0.05, **P < 0.01, and ***P < 0.001.

In order to assess the effects of CTEO on the viability of A549 lung cancer cells, after the treatment of CTEO at varied doses for 48 h, the cytotoxic activities were evaluated using a CCK-8 assay. As shown in (Fig 2A), it was observed that CTEO display significant anticancer effect with the increasing of drug concentrations. The IC_50_ of CTEO in A549 cells at 48 h was 48.38 μg/mL. The inhibition rate of the cells increases with the increase of the treatment time. When the treatment time was at 72 h, the IC_50_ value of CTEO in A549 cells was 30.7 μg/mL, as shown in (Fig 2B). However, the effects of CTEO on the human normal bronchial epithelial cells (HBE) were less inhibited and CTEO exhibited an IC_50_ value >100 μg/mL, as shown in (Fig 1C).

To further investigate the anti-cancer effect of CTEO, the colony formation assay was used. The number of colony formation was obviously decreased after treatment with 40 μg/mL or 60 μg/mL in A549 cells (Fig 2D). In addition, we detected the expression of proliferation cell nuclear antigen (PCNA) as a biomarker of proliferation. As shown in Fig 2E, the expression of PCNA was reduced in A549 cells after CTEO treatment, which was consistent with that of the CCK-8 assay and colony formation assay. Thus, CTEO appears to inhibit the proliferation of A549 cells.

### CTEO suppressed cell-cycle progression in A549 cancer cells

**Fig 3:**
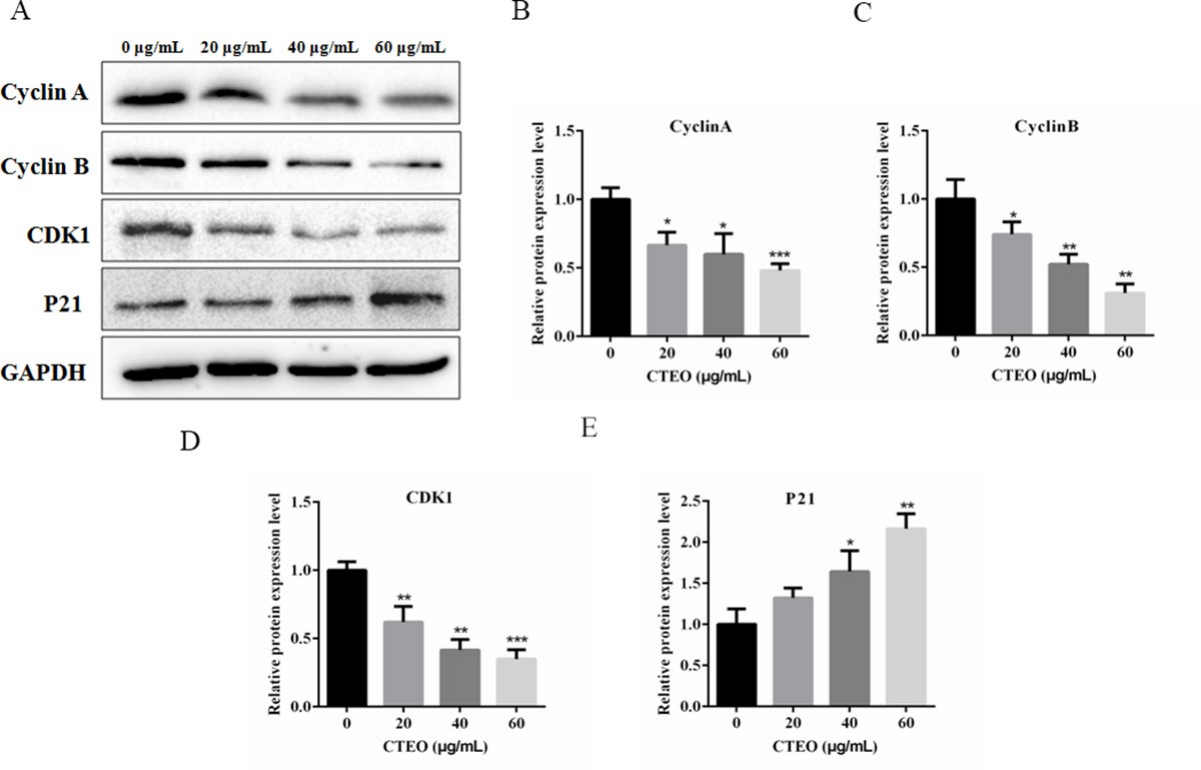
CTEO suppressed cell-cycle progression in A549 cells. A-E, The expression of cell cycle related protein cyclin A, cyclin B, CDK1 and P21 in A549 cells following 48 h of different concentrations CTEO treatment were detected by western blot analysis, and quantification. Values represent mean ± SD of three independent experiments. *P < 0.05, **P < 0.01, and ***P < 0.001.

To study the cellular mechanisms of how CTEO inhibits cell proliferations, we analyzed the changes in the expression levels of cell cycle factors related proteins in A549 cells. We assumed that CTEO treatment may impair the cyclin related proteins expressions in A549 cells. To verify this hypothesis, we detected the protein levels of cyclin proteins, such as cyclin A, cyclin B, CDK1 and P21 after treatment of different concentrations of CTEO for 48 h. The biochemical results showed that the expression levels of cyclin A, cyclin B and CDK1 were all reduced dramatically by CTEO treatment. However, P21, cyclin dependent kinase inhibitor, its expression significantly increased in a concentration-dependent manner after treated with CTEO (Fig 3A–3E). Taken together, all the results indicate that CTEO may decrease cyclin protein expressions at least partly, finally resulting in cell cycle arrest and cell growth inhibition.

### CTEO inhibits the migratory and invasive ability of A549 cells

**Fig 4:**
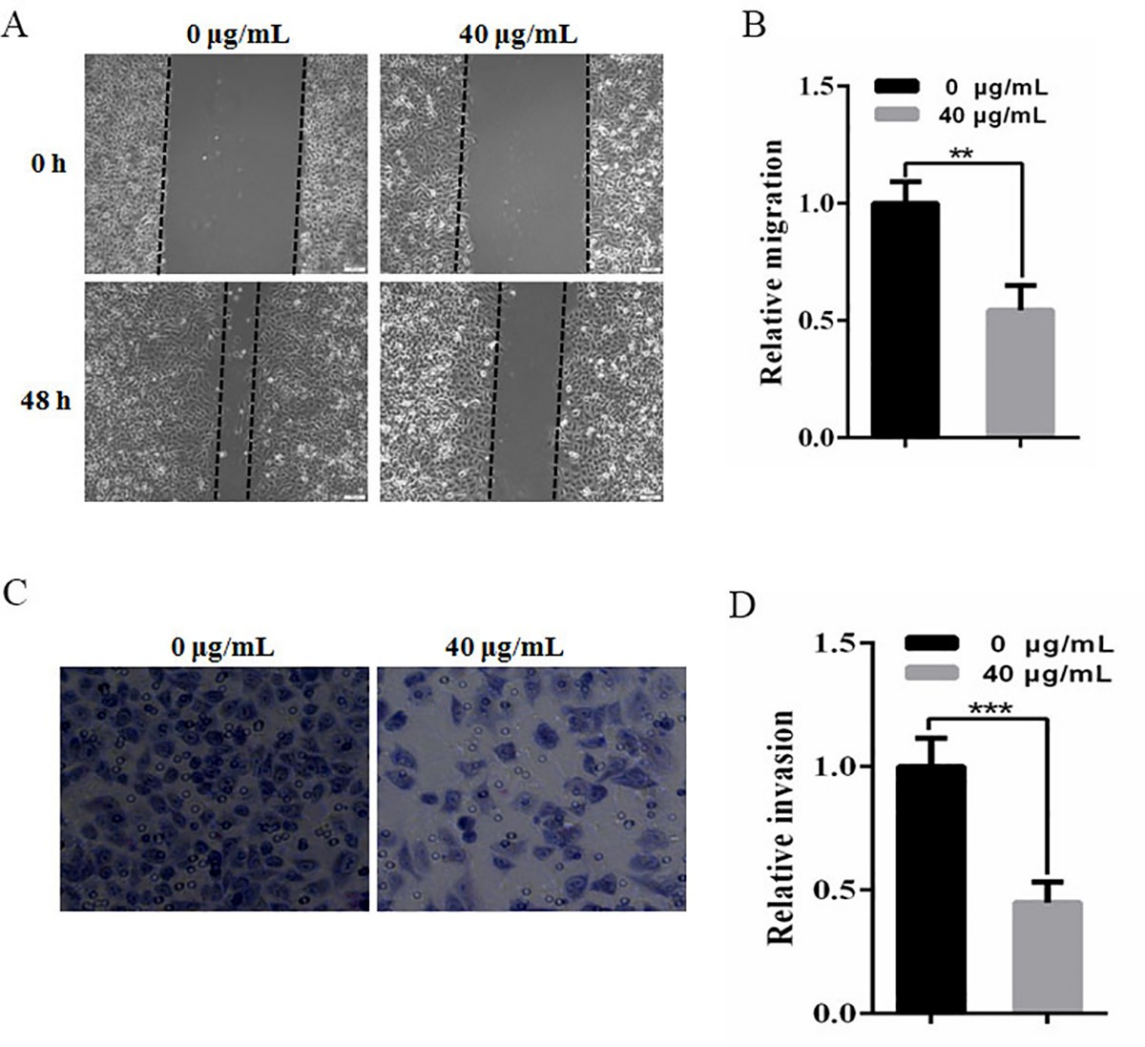
CTEO inhibits the migratory and invasive ability of A549 cells. A,B. Wound healing assay. A549 cells were treated with CTEO and artificial scratches were done with sterile 200 μL pipette. Photographs were taken after treatment. C,D. Transwell invasion chamber assay. The cells were treated the concentration indicated by CTEOfor 48 h. Cells invading the basement membrane were stained with Giemsa. Values represent mean ± SD of three independent experiments. *P < 0.05, **P < 0.01, ***P < 0.001 compared with the untreated control (dose 0).

To detect the effect of CTEO on migratory and invasive capabilities of A549 cells, wound scratch assay and transwell invasion assay were performed on A549 cells. It noted that, for A549 cells treated with 40 μg/mL CTEO, their wound healing rate was markedly decreased when compared with 0 μg/mL CTEO on the basis of these results. We also noted that, compared with A549 cells treated with 0 μg/mL CTEO, those treated with 40 μg/mL CTEO had dramatically lower invasive cell numbers, as shown in (Fig 4).

### CTEO inhibited the activation of FAK in A549 Cells

**Fig 5:**
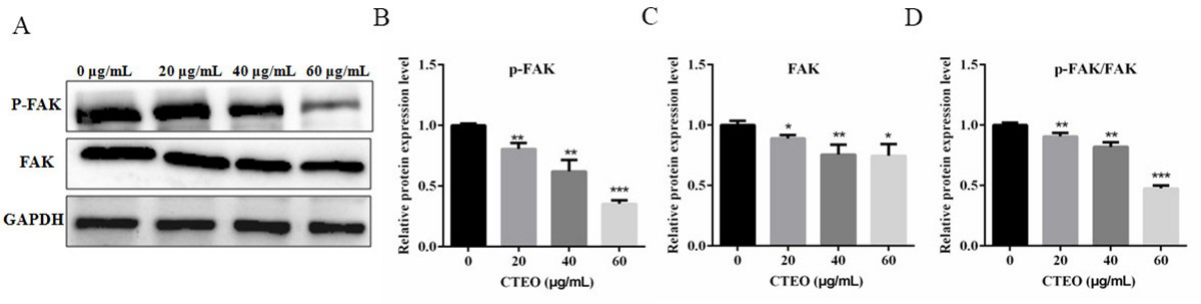
CTEO inhibited the activation of FAK in A549 cells. A-C. The expression of FAK and p-FAK protein in A549 cells following 48 h of different concentrations CTEO treatment were detected by western blot analysis, and quantification. C. The ratio of FAK to p-FAK protein expression was quantified analysis. Values represent mean ± SD of three independent experiments. *P < 0.05, **P < 0.01, and ***P < 0.001.

FAK plays a significant part in many types of cell events, such as proliferation, survival, apoptosis, migration and invasion. The above experiments have demonstrated that CTEO could significantly inhibit A549 cell migration and invasion. However, it still needs to continue to further verify whether CTEO can inhibit the metastasis and invasion of A549 cells through the FAK pathway. In the present study, A549 cells were treated with CTEO (0, 20, 40, 60 μg/mL) for 48 h. As shown in (Fig 5), the group which was treated with CTEO significantly decreased p-FAK level, but has no effect on the expression of FAK. These results suggested that reduced the expression of p-FAK may increase its inhibitory activity of cell migration and invasion.

### CTEO promoted A549 cells apoptosis in a dose-dependent manner

**Fig 6:**
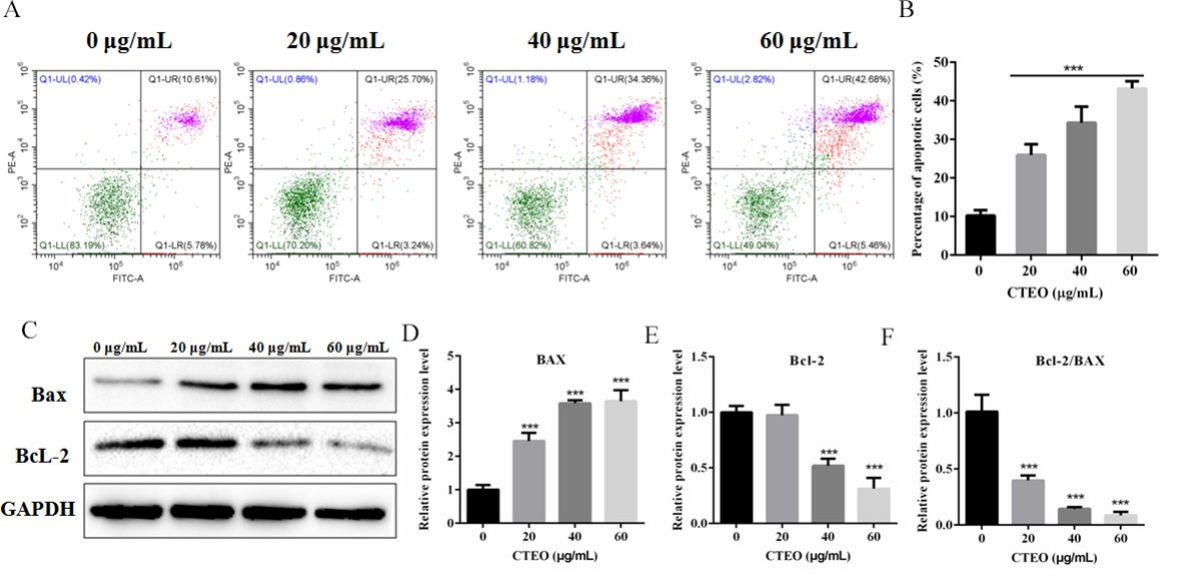
CTEO promoted A549 cells apoptosis in a dose-dependent manner. A, B. The apoptosis of A549 cells following 48 h of different concentrations CTEO treatment was detected by flow cytometry, and quantification. C-F. The expression of Bax and Bcl-2 protein in A549 cells following 48 h of different concentrations CTEO treatment were detected by western blot analysis, and quantification. Values represent mean ± SD of three independent experiments. *P < 0.05, **P < 0.01, and ***P < 0.001.

To determine whether CTEO could induce A549 cell apoptosis, the A549 cells were treated with varying concentrations of CTEO for 48 h and cells for changes in apoptotic markers was analyzed by flow cytometer. As shown in Fig 6A, the results of Annexin-V-FITC/PI exhibited that the apoptosis rate was significantly increased after the cells were treated with 20 μg/mL CTEO by the contrast of the 0 μg/mL group. And as the concentration increases, the number of cell apoptosis increases in a concentration-dependent manner (32.6% at 40 μg/mL, 42.5% at 60 μg/mL).

Subsequently, in order to study the mechanism that mediates CTEO-induced apoptosis, the expression of Bcl-2 and Bax in CTEO treatment of A549 cells was determined using a Western blot assay. The results demonstrated that the significant increase in the ratio of Bax/Bcl2 protein, the expression of the anti-apoptotic protein Bcl-2 was decreased, while the expression of the pro-apoptotic proteins Bax were increased in CTEO group compared with the corresponding values in control group. As shown in (Fig 6C); P<0.05. The results indicated that CTEO may promote the apoptosis of A549 cells.

### CTEO induces mitochondrial dysfunction in A549 cells

**Fig 7:**
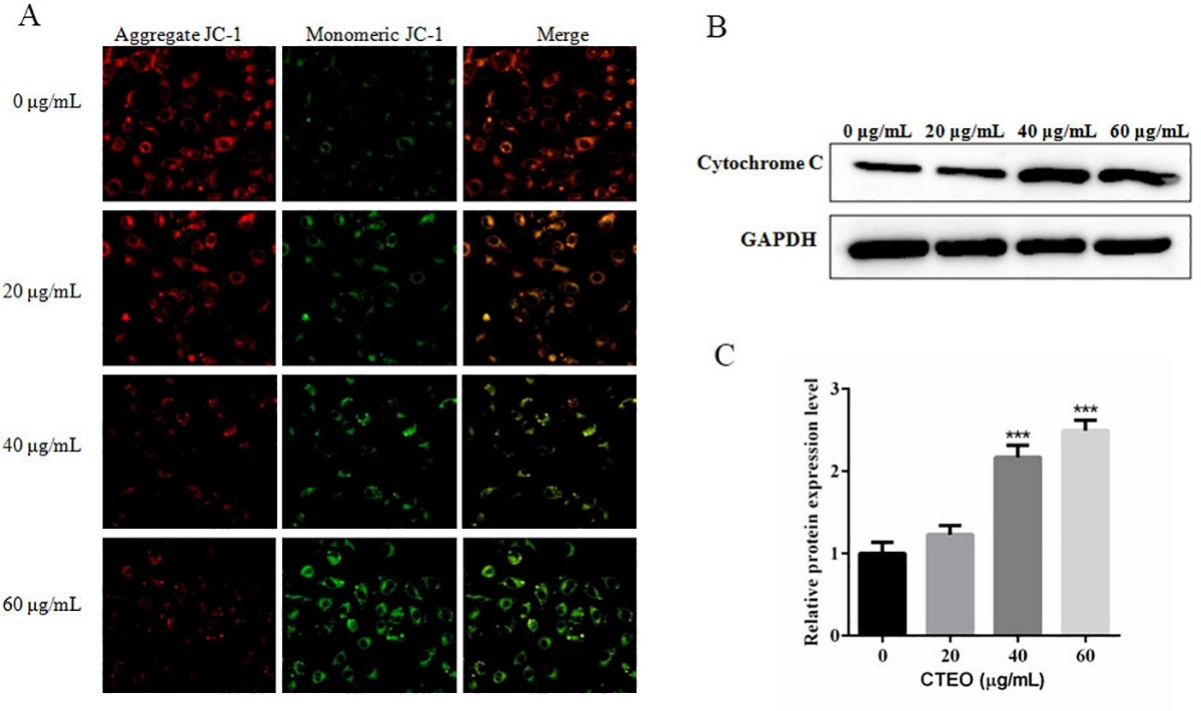
CTEO induces mitochondrial dysfunction in A549 cells. A. The change of mitochondrial membrane potential in A549 cells following 48 h of different concentrations CTEO treatment was detected by JC-1 staining analysis. B, C. The expression of cytochrome C protein in A549 cells following 48 h of different concentrations CTEO treatment were detected by western blot analysis, and quantification. Values represent mean ± SD of three independent experiments. *P < 0.05, **P < 0.01, and ***P < 0.001.

As we all know, the mitochondria play an important part in the regulation of cell apoptosis. Mitochondrial membrane potential collapse is an early feature of cell apoptosis. JC-1 staining was used to assess the loss of mitochondrial membrane potential by fluorescence microscope and western blotting to evaluate related protein expression in A549 cells. As shown in Fig 7A, the results certified that with the increase of CTEO (0, 20, 40 and 60 μg/mL), the green fluorescence gradually increased while the red fluorescence gradually decreased, indicating that the mitochondrial membrane potential gradually decreased. Subsequently, we also examined the changes of cytochrome C in A549 cells. The results demonstrated that the protein expression level of Cytochrome C in the cytoplasm gradually increased with the increase of CTEO concentration, as shown in Fig 7B.

### CTEO induces apoptosis via caspase-mediated pathways in A549 cells

**Fig 8:**
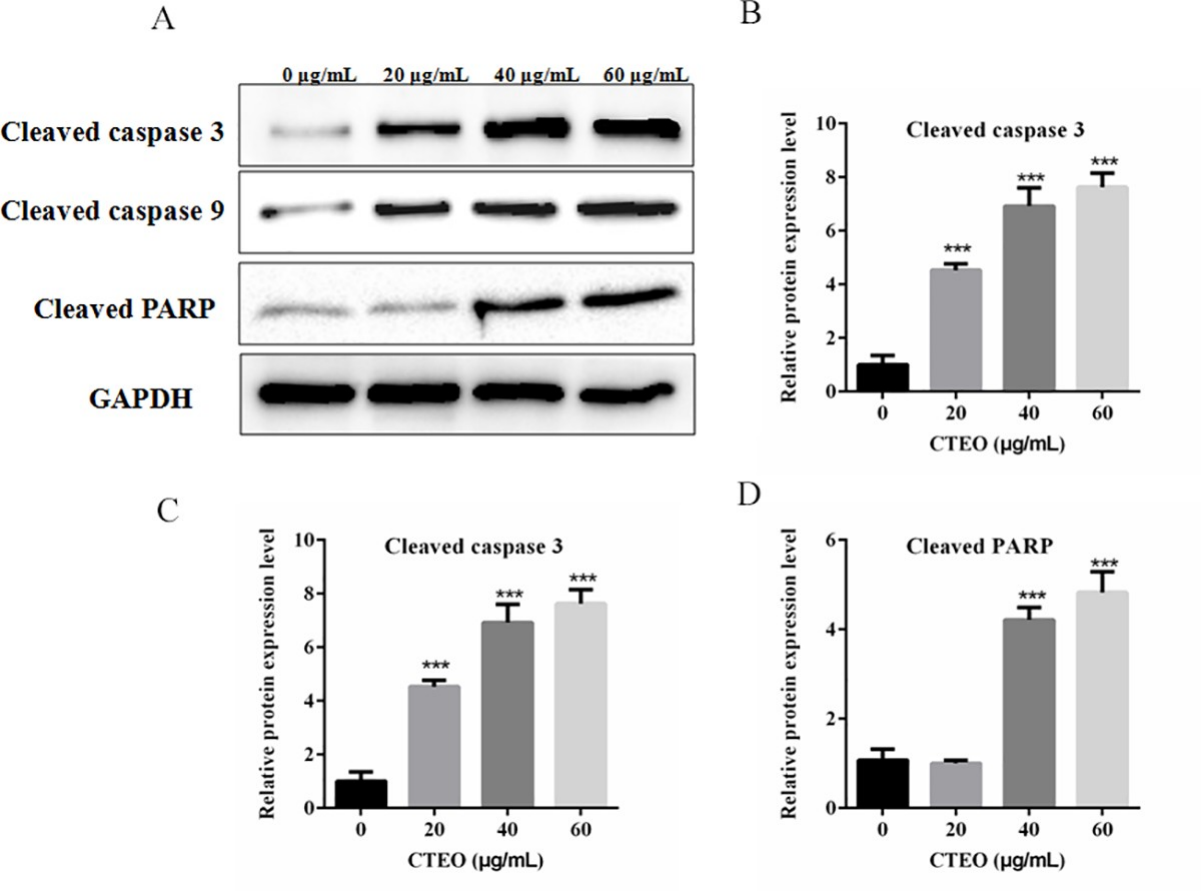
CTEO induces apoptosis via caspase-mediated pathways in A549 cells. A-D. The expression of apoptosis-related protein in A549 cells following 48 h of different concentrations CTEO treatment were detected by western blot analysis, and quantification. Values represent mean ± SD of three independent experiments. *P < 0.05, **P < 0.01, and ***P < 0.001.

Cysteine proteases can transmit apoptotic signals in the proteolytic cascade, after caspase cleaving, it can activate other caspase enzymes and subsequently degrades cellular targets that cause cell death. The above results indicated that CTEO treatment led to reduce in the ratio of Bcl2/ Bax protein, and an increase in the expression level of Cytochrome C in the cytoplasm. To investigate the potential mechanism of cell apoptosis and whether CTEO induce apoptosis in A549 cells via a caspase-mediated pathway. We assessed the apoptotic markers in A549 cells by gradient CTEO treatment using Western blot analysis. As shown in Fig 8A, the level of cleaved caspase-3, cleaved caspase-9 were markedly up-regulated by CTEO in the A549 cells, and the level of cleaved PARP was also significantly increased compared with the control.

## Discussions

Lung cancer is one of the most common malignancies in the world, and its high incidence and recurrence rate are the leading causes of cancer death [24]. Uncontrolled mitosis and rapid migration and migration of cancer cells are hallmarks of cancer, which are associated with poor prognosis in many tumors, such as lung, breast and osteosarcoma [25, 26]. Thus, targeting inhibition of cell proliferation or promoting apoptosis can provide clues to cancer treatment. Previous reports showed that there are abundant of linoleic acid, oleic acid and eicosenoic acid in the seeds oil extracted with petroleum from Chinese *C*. *tiglium*. Instead, carcinogenic and anti-HIV-1 active ingredient may be very rare in the sample, and it is difficult to obtain by conventional extraction and separation method [27]. Therefore, in this study, the essential oil of *C*. *tiglium* was obtained by alternative technique (SFE) firstly for the further research.

One study has previously reported on the chemical composition of CTEO, and from which 17 fat acid components were identified. The main components were linoleic acid, oleic acid, and eicosenoic acid in ethy-esterified sample, occupying 77.33% of the total oil. In our present study, the mainly chemotypes of identified compounds are also fatty acid, these results are consistent with that previously reported [28]. However, many volatile compounds that had not been reported in previous studies were identified, such as 17-Octadecynoic acid (36.73%), Tetradecanoic acid (8.49%), *n*-Hexadecanoic acid (6.45%), Undecanoic acid (2.54%) etc.. In addition, this alternative technique (SFE) generates very more chemical components (28 compounds were identified) than those obtained by petroleum extraction, which will benefit for further processing of the *C*. *tiglium* plants.

As described in a previous paper [29]. To study the cytotoxicity of essential oils, an IC_50_ value which less than 50 μg/mL represents a strong cytotoxic activity. In addition, IC_50_ values between 50–100, 100–200 and 200–300 μg/mL represent moderate, weak and very weak cytotoxicity, respectively. With this in mind, the IC_50_ value of CTEO in this study was 48.38 μg/mL, indicating strong cytotoxicity against A549 cells (Fig 2). Therefore, the essential oil obtained from *C*. *tiglium* has great potential as a source of natural anti-cancer drugs.

In the present study, CTEO showed significant antitumor activity attributable to the presence of fatty acids, which accounted for 78.48% of the total oil (Table 1). Previous studies have demonstrated that fatty acids have significant anti-tumor effects [30]. Butyrate, histone deacetylase inhibitor, is a typical short chain fatty acid, which have significant anti-cancer activity against colon cancer cell line HCT116. Correlation analysis showed that butyrate treatment significantly inhibited the proliferation of HCT116 cells and increased the ratio of Bax/Bcl-2 protein to induce apoptosis [31]. Nitro-Fatty Fatty Acid (NFA) is an endogenous lipid mediator. It has been found that NFA exerts a strong anti-cancer effect on colorectal cancer (CRC) cells and inhibits the viability of CRC cells (HCT-116 and HT-29). The mitochondrial endogenous apoptotic pathway induces caspase-dependent apoptosis. Therefore, the total oil is selected to study the relevant mechanisms of the observed effects.

PCNA, as a biomarker of proliferation, the expression was down-regulated in A549 cells after CTEO treatments (Fig 2). The inhibitory effect of CTEO was further confirmed by colony formation assay. These results indicate that CTEO has an inhibitory effect on A549 cell growth *in vitro*. Cell proliferation is dependent on the progression of the cell cycle. Another very important mechanism used by anticancer agents is cell cycle arrest. If the cancer cell were arrested in any phase of the cell cycle, they are unable to complete cell division and tumor growth is inhibited [30]. Moreover, the proliferative inhibitory effects of CTEO on A549 cells may be at least partly dependent on cyclin protein expressions, because it was noted that CTEO treatment would inhibit cyclin protein expression (Fig 3). Thus, the reduced cyclin protein expressions may be responsive to the cell cycle arrest and consequently apoptosis.

Cancer cell metastasis is associated with various steps, including adhesion of cancer cells to ECM, degradation of the basement membrane to allow tumor cells to migrate and invade peripheral tissues [32, 33]. Many types of cell adhesion molecules play important roles in different steps of metastatic events [34]. Focal adhesion kinase (FAK) is a major mediator of signal transduction through cells and extracellular matrices and plays an important role in cell proliferation, survival, apoptosis, migration and invasion [35, 36]. FAK is phosphorylated when activated. Tyr 397 (Y397) is the main phosphorylated site. By binding with other kinase domains, FAK activates downstream signaling pathways, regulates cell migration and induces apoptosis [33]. The elevated FAK and phospho-FAK Tyr397 are associated with the development, invasion and metastasis of several tumors [37]. Previous studies [38] have shown that treatment of tumor cells with FAK-specific inhibitors could inhibit tumor cell proliferation, migration and invasion. However, in our results, CTEO was able to significantly inhibit the migration and invasion (Fig 4), and decrease p-FAK protein levels in a dose-dependent manner in A549 cells (Fig 5).

Apoptosis inhibits cancer cell survival and has been identified as a central mechanism for the induction of cell death by cytostatic drugs. Cytostatic drugs can cause apoptosis through a variety of pathways, including activation of death receptor/ligand expression, changes in mitochondrial membrane potential, ROS production, DNA damage etc [39]. Most anti-cancer drugs could promote tumor cell apoptosis by up-regulating the ratio of Bax/Bcl-2 [40]. Consistent with previous studies, our results find that CTEO could increase the protein expression of Bax and reduce the levels of Bcl-2 in A549 cells in a dose-dependent manner (Fig 6). The increased Bax/Bcl-2 ratio could cause a decrease in the mitochondrial membrane potential, resulting in the release of cytochrome c into the cytosol. Decreased mitochondrial membrane potential is an early phenomenon of tumor cell apoptosis. The released cytochrome c activates Caspase-9, and activated caspase-9 further activates caspase-3. Thus the caspase cascade is activated and PARP is cleaved to cause apoptosis [41]. Our findings revealed that CTEO caused a dramatic loss of mitochondrial membrane potential and accumulation of cleaved caspase-3、cleaved caspase-9 and cleaved PARP (Figs 7 and 8). The above results indicate that CTEO-induced caspase cascade activation together with subsequent PARP cleavage implying CTEO-mediated apoptosis occurs via the intrinsic pathway.

## Conclusion

**Fig 9:**
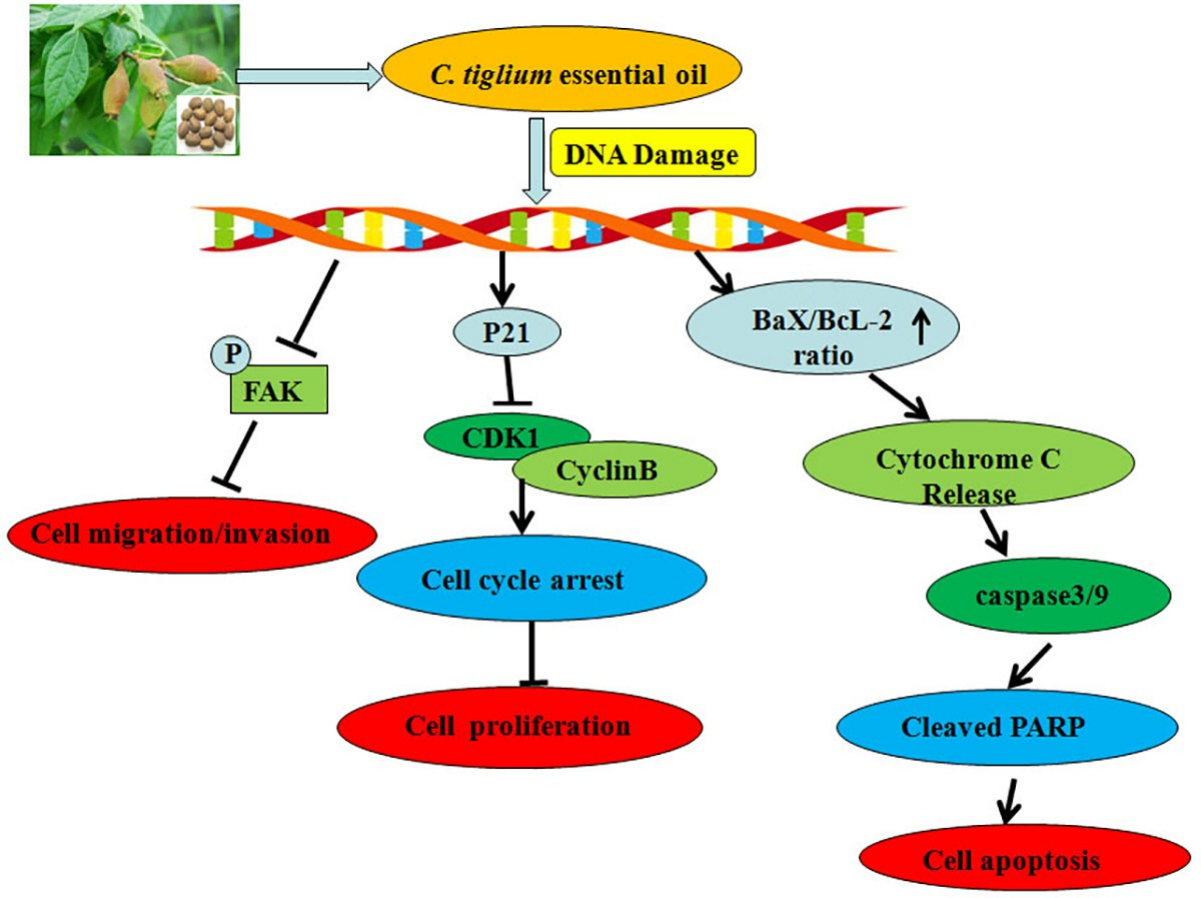
Proposed mechanisms of the antitumor effects of CTEO on lung cancer cells. CTEO depresses cell growth, invasion, migration and induces cell apoptosis in A549cells via (1) decreasing expression of Proliferation factors;(2) inhibiting invasion and migration factors; and (3) enhancing expression of pro-apoptotic factors.

Taking these results into consideration, we propose that the anti-cancer effect of CTEO on A549 cells is partly through decreasing expression of proliferation factors, inhibiting invasion and migration factors, and enhancing expression of pro-apoptotic factors, as shown in Fig 9. Natural resources capable of inducing tumor cell cycle arrest and apoptosis are expected to be candidates for anticancer drugs [42]. Therefore, these evidences suggested that CTEO may be a promising and future supplement in the investigation of novel chemopreventive or chemotherapeutic agents for human lung cancers and may be considered for further clinical studies in drug development. These findings should benefit the development of *C*. *tiglium* industry.

## Supporting information

S1 Fig Composition of CTEO on HP-5MS column. (TIF)

S2 Fig Composition of C7-C40 alkanes on HP-5MS column. (TIF)

S1 Raw Images All replicates PCNA and GAPDH blots. S1-raw-images represents western blot analysis shown in Fig 2. Lanes 1–4 represent 0. 20. 40. 60 μg/mL group.
(TIF)

S2 Raw Images All replicates cyclin A. cyclin B. CDK1. P21 and GAPDH blots. S2-raw-images represents western blot analysis shown in Fig 3. Lanes 1–4 represent 0. 20. 40. 60 μg/mL group.
(TIF)

S3 Raw Images All replicates FAK. P-FAK and GAPDH blots. S3-raw-images represents western blot analysis shown in Fig 5. Lanes 1–4 represent 0. 20. 40. 60 μg/mL group.
(TIF)

S4 Raw Images All replicates BAX. Bcl-2 and GAPDH blots. S4-raw-images represents western blot analysis shown in Fig 6. Lanes 1–4 represent 0. 20. 40. 60 μg/mL group.
(TIF)

S5 Raw Images All replicates Cytochrome C and GAPDH blots. S5-raw-images represents western blot analysis shown in Fig 7. Lanes 1–4 represent 0. 20. 40. 60 μg/mL group.
(TIF)

S6 Raw Images All replicates cleaved caspase 3. Cleaved caspase 9. Cleaved PARP and GAPDH blots. S6-raw-images represents western blot analysis shown in Fig 8. Lanes 1–4 represent 0. 20. 40. 60 μg/mL group.
(TIF)
