# Supplementary figures and images for: Croton tiglium essential oil compounds have anti-proliferative and pro-apoptotic effects in A549 lung cancer cell lines
Source: PLoS One. 2020 May 1;15(5):e0231437. doi: 10.1371/journal.pone.0231437 (PMC7194401; doi:10.1371/journal.pone.0231437)

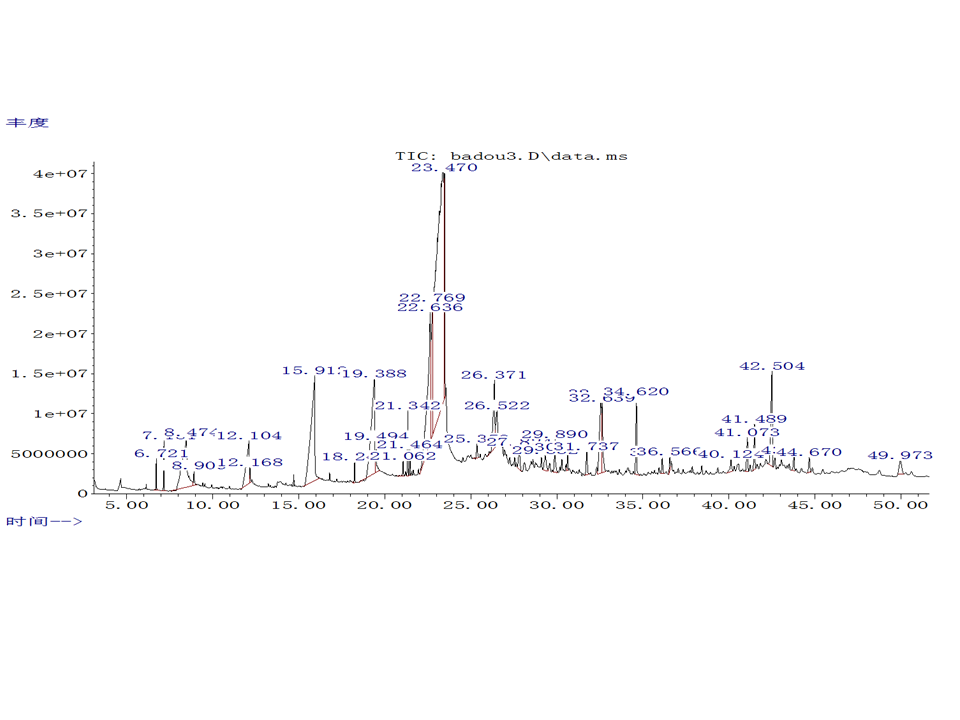

Supplement: S1 Fig — (TIF) [file pone.0231437.s001.tif]

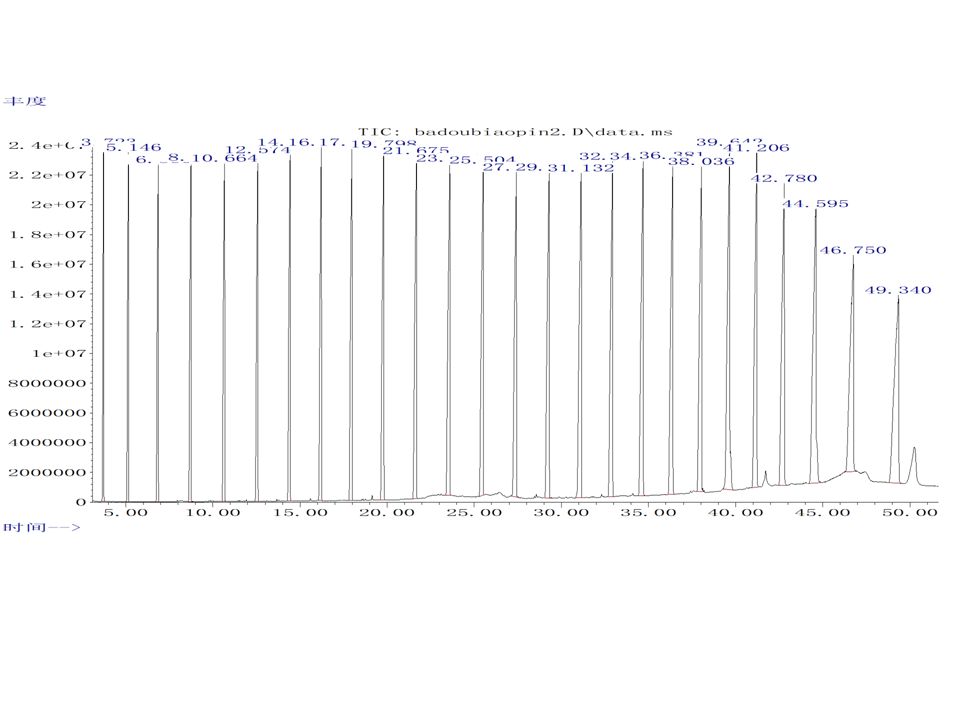

Supplement: S2 Fig — (TIF) [file pone.0231437.s002.tif]

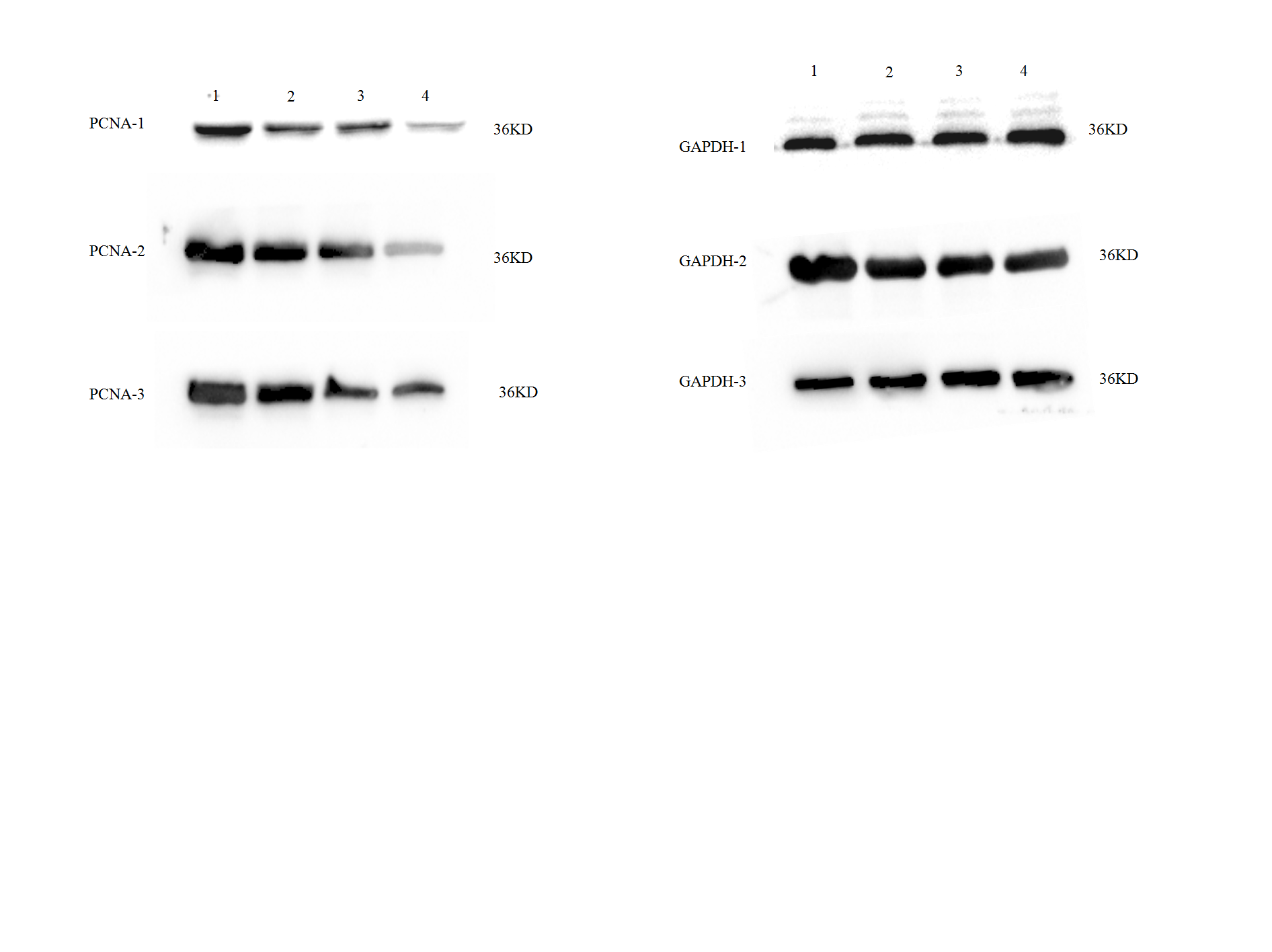

Supplement: S1 Raw Images — S1-raw-images represents western blot analysis shown in Fig 2. Lanes 1–4 represent 0. 20. 40. 60 μg/mL group. (TIF) [file pone.0231437.s003.tif]

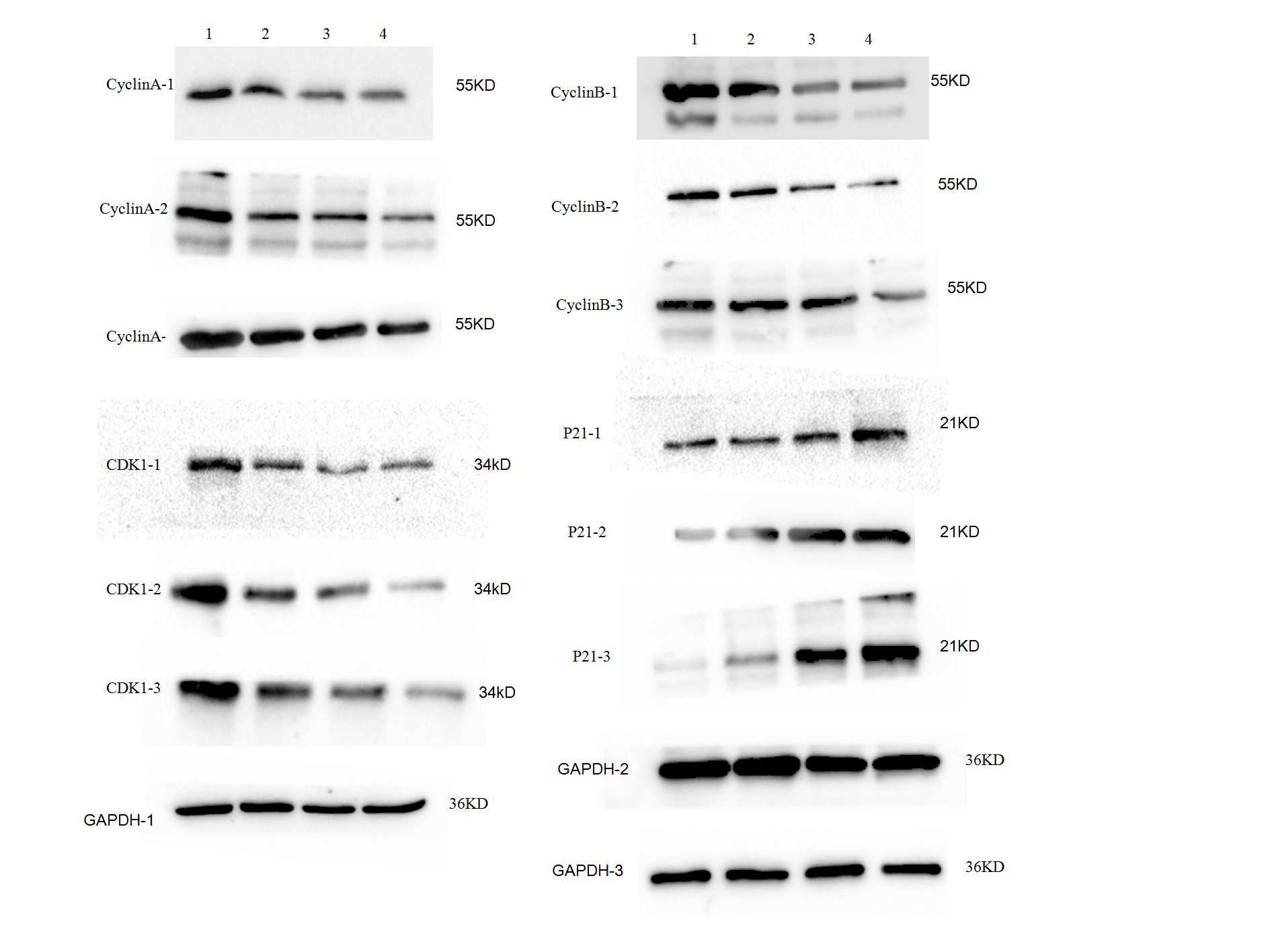

Supplement: S2 Raw Images — All replicates cyclin A. cyclin B. CDK1. P21 and GAPDH blots. S2-raw-images represents western blot analysis shown in Fig 3. Lanes 1–4 represent 0. 20. 40. 60 μg/mL group. (TIF) [file pone.0231437.s004.tif]

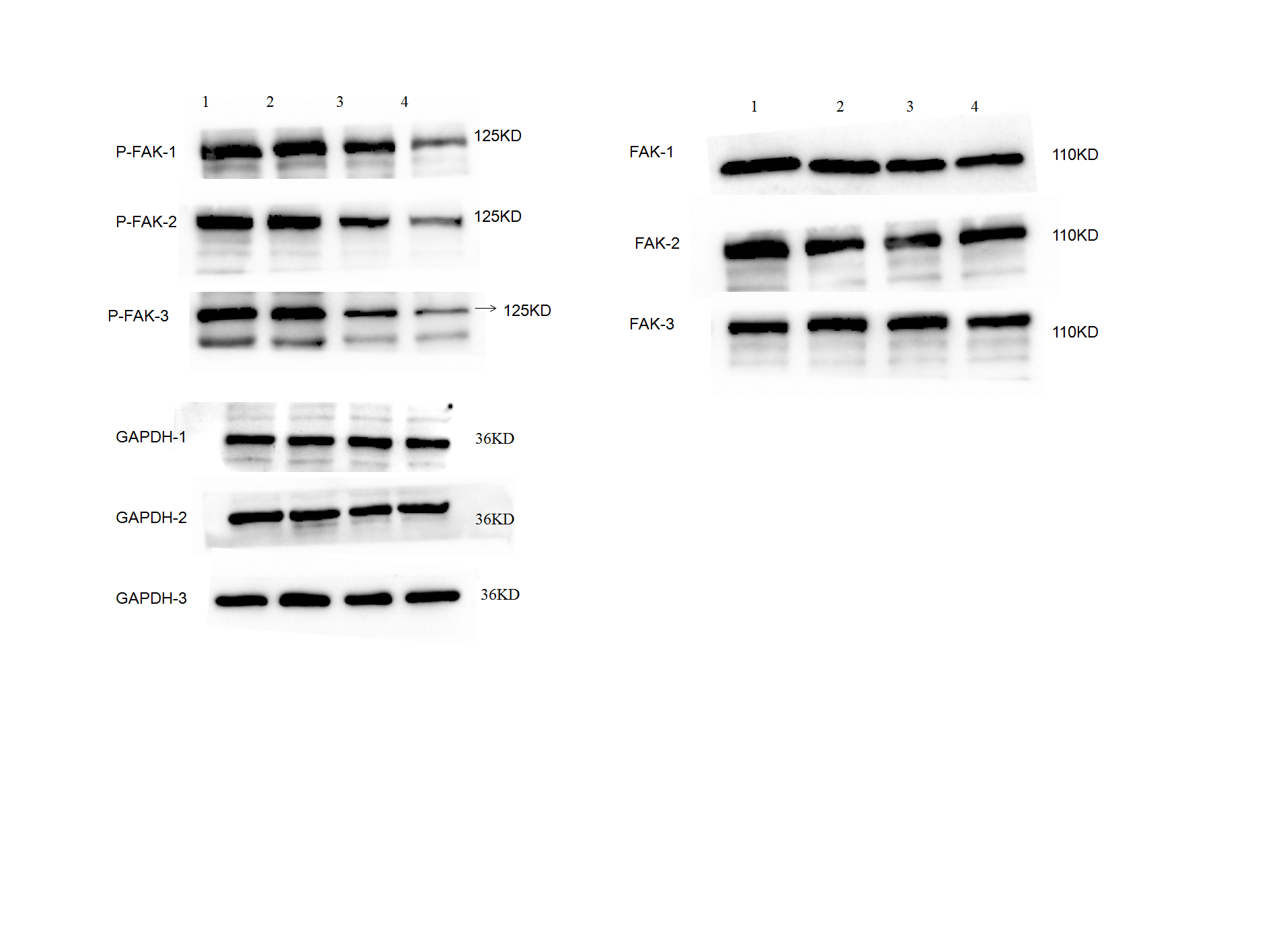

Supplement: S3 Raw Images — P-FAK and GAPDH blots. S3-raw-images represents western blot analysis shown in Fig 5. Lanes 1–4 represent 0. 20. 40. 60 μg/mL group. (TIF) [file pone.0231437.s005.tif]

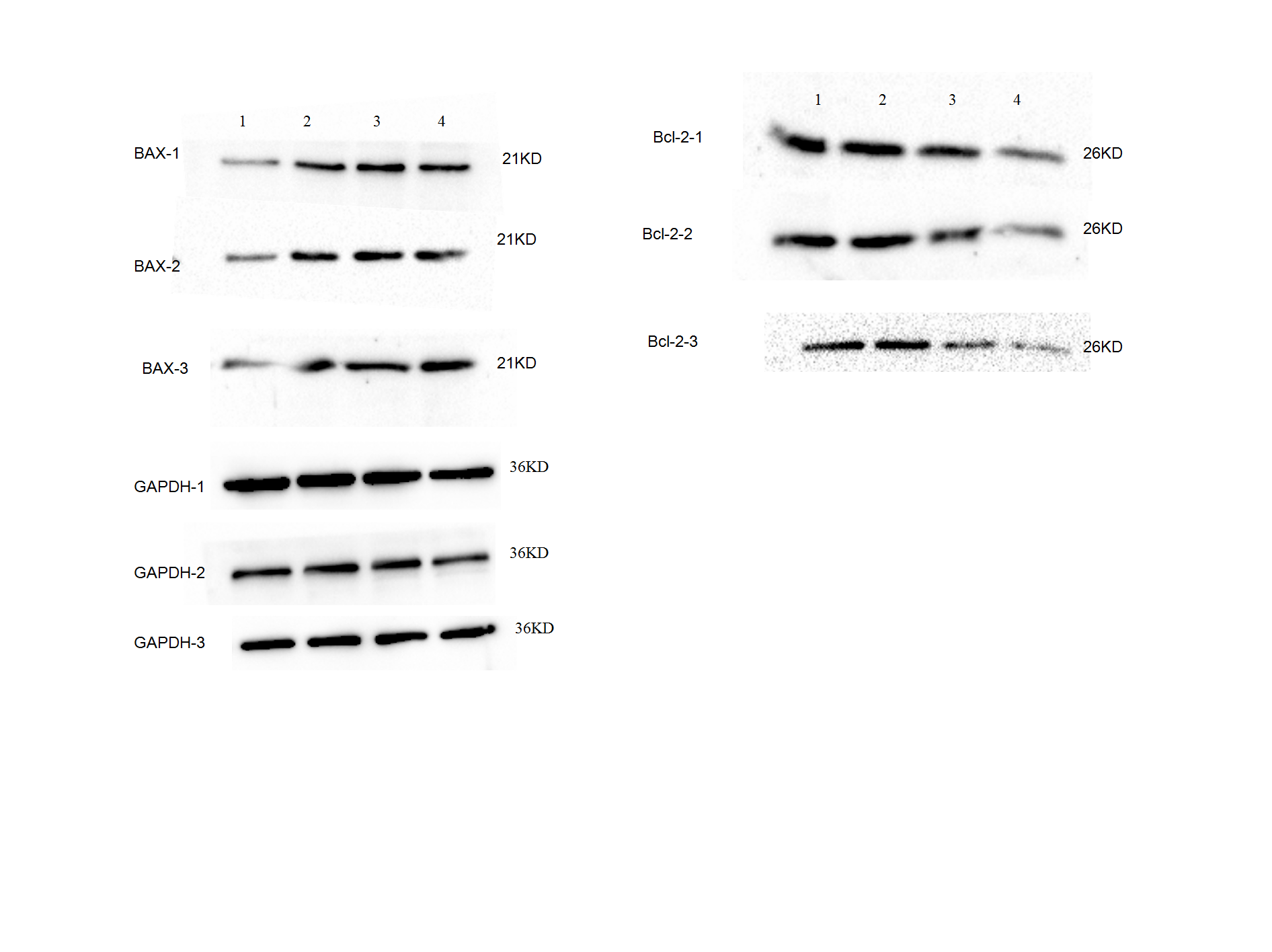

Supplement: S4 Raw Images — Bcl-2 and GAPDH blots. S4-raw-images represents western blot analysis shown in Fig 6. Lanes 1–4 represent 0. 20. 40. 60 μg/mL group. (TIF) [file pone.0231437.s006.tif]

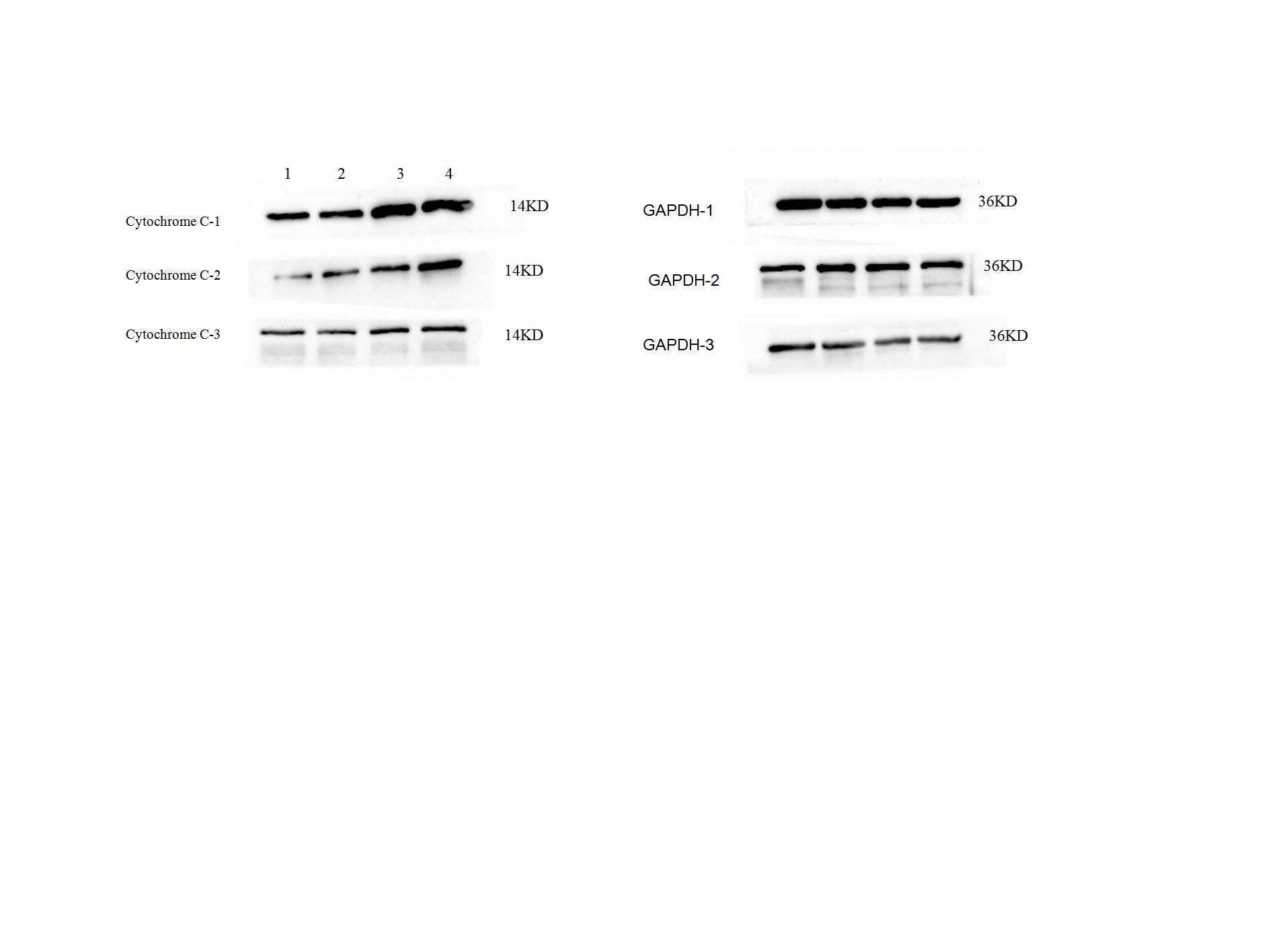

Supplement: S5 Raw Images — S5-raw-images represents western blot analysis shown in Fig 7. Lanes 1–4 represent 0. 20. 40. 60 μg/mL group. (TIF) [file pone.0231437.s007.tif]

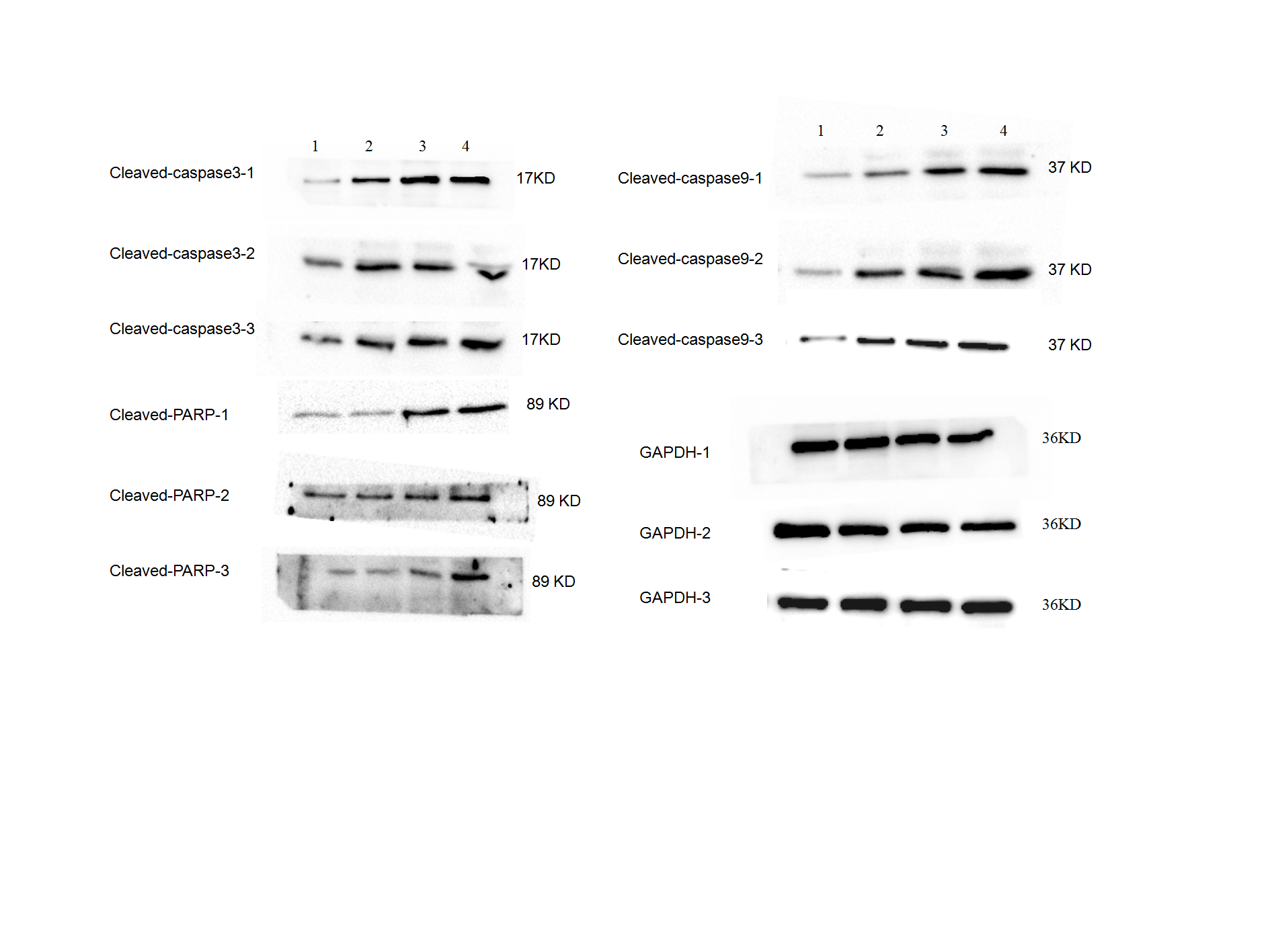

Supplement: S6 Raw Images — Cleaved caspase 9. Cleaved PARP and GAPDH blots. S6-raw-images represents western blot analysis shown in Fig 8. Lanes 1–4 represent 0. 20. 40. 60 μg/mL group. (TIF) [file pone.0231437.s008.tif]
